# Study on the Bactericidal Mechanism of Atmospheric-Pressure Low-Temperature Plasma against *Escherichia coli* and Its Application in Fresh-Cut Cucumbers

**DOI:** 10.3390/molecules23040975

**Published:** 2018-04-22

**Authors:** Yan Sun, Zhiwei Zhang, Shiqing Wang

**Affiliations:** College of Food Science and Engineering, Qingdao Agricultural University, Qingdao 266109, China; 18363976348@163.com

**Keywords:** atmospheric-pressure low-temperature plasma (APLTP), *E. coli*, bactericidal mechanism, fresh-cut cucumber

## Abstract

Atmospheric-pressure low-temperature plasma (APLTP) was used to study the bactericidal mechanism against *Escherichia coli* (*E. coli*) and its application in the sterilization of fresh-cut cucumbers. The morphological changes of *E. coli* cells subjected to APLTP were observed by scanning electron microscopy (SEM). Cell death was evaluated by fluorescence microscopy (FM). Cell membrane permeability was measured by conductivity changes, and the amount of soluble protein leakage in the bacterial supernatant was determined by measurement of protein concentration. Additionally, the effects of APLTP on the physicochemical and sensory quality of fresh-cut cucumber were studied by assessing the changes of moisture content, soluble solid content (SSC), pH value, color, relative conductivity, malondialdehyde (MDA) level, vitamin C (Vc) content, aroma composition and microstructure. The results showed that the *E. coli* cell morphology was changed due to the charged particles and active components produced by APLTP. The *E. coli* cell wall and cell membrane ruptured, cell content leaked out, cells lost the ability to reproduce and self-replicate, and the function of cell metabolism was directly affected and led to *E. coli* inactivation. In addition, there was no significant effect on physicochemical properties and sensory quality of fresh-cut cucumbers.

## 1. Introduction

Plasma is an ionized gas, which consists of an assembly of ions, electrons and neutral particles (atoms or molecules). The charges of ions and electrons are equal and show overall neutrality. It is a kind of ionized state composed of charged particles, and is called the fourth state of matter besides the three states of solid, liquid and gas. Plasma sterilization and decontamination is widely used in biology, medicine and food security [1,2,3].

Atmospheric-pressure low-temperature plasma (APLTP) is an emerging nonthermal technology that can potentially decontaminate the surfaces of fresh produce. This antimicrobial intervention offers the advantages of being chemical free, water free, low temperature, highly efficient, nonpolluting and nontoxic. In addition, it is able to operate openly and continuously at atmospheric pressure when used as a new green sterilization technology. Plasma sterilization is widely used in food applications due to its ability to maintain the color, smell, taste and shape of foods to the maximum extent [4,5,6,7,8,9].

Criter [10] used plasma to treat *Escherichia coli* (*E. coli*) O157:H7, *Salmonella* and *Listeria monocytogenes* on the surface of apple, melon and lettuce, and the number of colonies in all samples was significantly reduced. It was also reported that when *E. coli*, *Saccharomyces cerevisiae*, *Pantoea agglomerans* and *Gluconacetobacter* were inoculated on the peel of mango and lemon, cold plasma treatment can effectively kill microorganisms inoculated onto fresh produce [11]. Moreover, when *Salmonella* and *E. coli* O157:H7 were inoculated on apple peel and treated with plasma generated by sliding arc medium, the *Salmonella* and *E. coli* decreased by 2.9–3.7 log CFU/mL and 3.4–3.6 log CFU/mL, respectively [12]. In addition, Stefano [13] found that the *L. monocytogenes* and *E. coli* O157:H7 concentrations on the mango surface decreased by 2.5 log CFU/g after only 30 s treatment with cold-source plasma.

It has been demonstrated that highly efficient sterilization of various bacteria can be achieved at low temperature by nonthermal plasmas, such as dielectric barrier discharge (DBD) [14], glow discharge [15], corona discharge [16] and plasma jet [17]. Among these plasma sources, the plasma jet is believed to be a feasible one because it generates the plasma outside the discharge region and in ambient air, which expands its application scope [18,19]. Low-temperature plasma generation is a very complex physical and chemical process, and can produce ultraviolet (UV) radiation, charged particles, active ingredients and other bactericidal agents. Transient electric fields of quite large amplitude are likely to be active in cell permeabilization or poration [20,21,22]. Active ingredients mainly include atoms in the excited state, metastable atoms, and oxides and nitrides with active chemical properties, able to induce the change of cell membrane permeability [23,24,25].

The role of plasma in the organism is mainly achieved through the combined effect of these bactericidal agents in the organism at the molecular level. Currently, further studies are needed to determine which one of these factors plays a leading bactericidal role and how it works at the molecular level. 

In this study, APLTP was used to treat a bacterial suspension of *E. coli,* which was used as the model microorganism. The changes of the morphology of cells treated with APLTP were examined by scanning electron microscopy (SEM). Cell death was detected by fluorescent microscopy (FM). Cell membrane permeability was measured by conductivity changes, and the amount of soluble protein leakage in the bacterial supernatant was determined by measurement of the protein concentration. Additionally, APLTP was applied to fresh-cut cucumber, and the physicochemical properties and sensory quality of fresh-cut cucumber before and after treatment were compared to verify the practicability of APLTP as a sterilization method for cucumber and similar food products.

## 2. Results

### 2.1. The Bactericidal Mechanism

#### 2.1.1. SEM Observation

**Figure 1:**
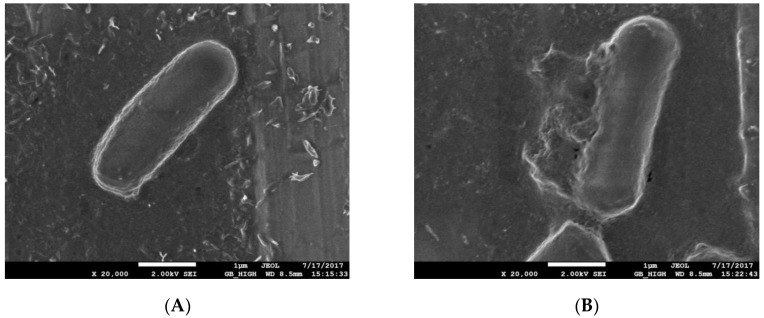
Scanning electron microscopy (SEM) images of *E. coli.* (**A**) Control *E. coli*; (**B**) Treated *E. coli.*

The SEM images of the normal cultured *E. coli* and plasma-treated *E. coli* bacterial morphology are shown in Figure 1.

The SEM images reveal that the control nontreated *E. coli* cells were blunt round at both ends, without *Bacillus* spores, and had a full, smooth surface (Figure 1A). After the plasma treatment, the morphology of the *E. coli* cells (Figure 1B) was irregular, and some of the surface cells were dented, wrinkled or broken. Additionally, the boundary of the cell wall in some cells was unclear, some of the cells appeared leaking, and a large amount of cell debris was observed. Thus, plasma treatment destroyed the cell membrane of *E. coli*, caused its contents to flow out and resulted in its inactivation.

#### 2.1.2. FM Observation

**Figure 2:**
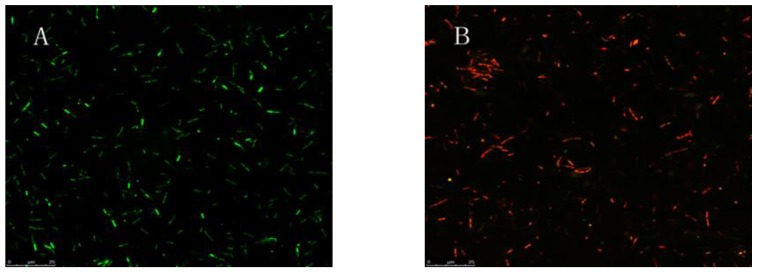
Fluorescent microscopy (FM) images of *E. coli.* (**A**) Control *E. coli*; (**B**) Plasma-treated *E. coli.*

The plasma-treated *E. coli* was double-stained with acridine orange/propidium iodide (AO/PI) and observed by fluorescence microscopy. AO permeates the normal cell membrane, and fluorescently stains the nucleus green or yellow–green. PI is a red fluorescent nucleic acid intercalating dye that cannot permeate the membrane of living cells, and thus can only stain the cells with disrupted cell membranes, making it useful to differentiate dead cells and healthy cells. The FM images showed that the control *E. coli* cells (Figure 2A) showed green fluorescence, while the plasma-treated *E. coli* (Figure 2B) showed red fluorescence. This result indicates that plasma treatment gradually increased the permeability of cell walls and cell membranes, allowing the PI to enter the cell and bind to genomic DNA. Accordingly, this result revealed that all of the plasma-treated *E. coli* were killed, which is consistent with the results of previous experiments on sterilization of fresh-cut cucumber.

#### 2.1.3. Evaluation of Conductivity

**Figure 3:**
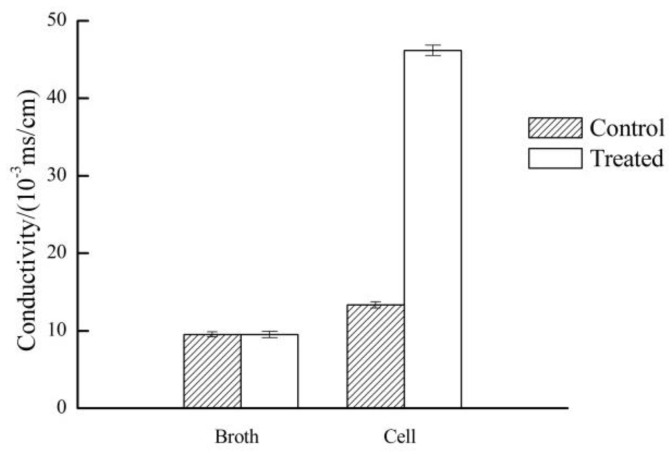
Effect of APLTP treatment on *E. coli* conductivity.

The bacterial cell membrane forms a barrier that allows the passage of small molecules such as K^+^, Na^+^ and H^+^. These small molecules play a crucial role in maintaining membrane function, enzyme activity and normal metabolism in the bacterial cells. The infiltration of small molecules is controlled by the membrane structure and composition [26]. The change of conductivity of culture medium can reflect the change of cell membrane permeability. The results presented in Figure 3 reveal that the conductivity of LB broth had no significant difference, but the conductivity of the plasma-treated *E. coli* cells increased significantly, indicating that the ionic homeostasis was destroyed due to the effect of the APLTP treatment on the bacterial cell membrane. It is thought that due to major electrolyte leakage, the cell membrane barrier was disrupted, thus affecting cell metabolism and eventually leading to *E. coli* cell death.

#### 2.1.4. Determination of Protein Concentration

**Figure 4:**
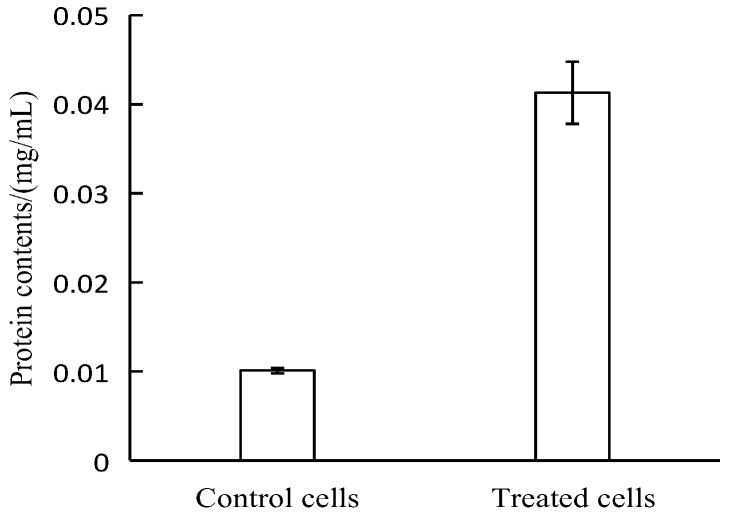
Effect of APLTP on *E. coli* protein content.

Proteins are important structural components of the cell membrane and cytoplasm [27]. The release of proteins indicates that cell membrane integrity has been seriously compromised. The results of the measurement of protein concentration are shown in Figure 4. After APLTP treatment, the protein content in the *E. coli* suspension increased by 5.6 times. Thus, according to this result, treatment by APLTP damaged the cell membrane, which led to the leakage of a large amount of protein and affected the normal cell growth.

#### 2.1.5. Optical Emission Spectrum

**Figure 5:**
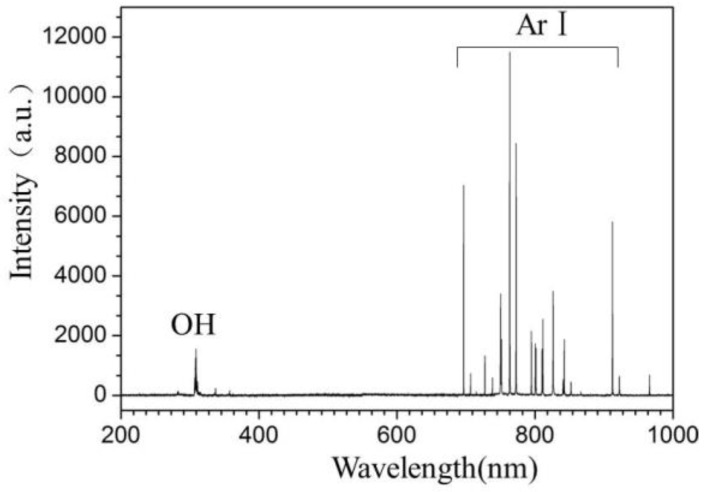
Optical emission spectrum of APLTP.

Optical emission spectroscopy of APLTP is shown in Figure 5. The most prominent spectral features are neutral argon atomic lines in the region from 700 to 900 nm, and excited OH (307–310 nm). The quartz glass was placed above the fresh-cut cucumbers to study the effect of UV photons generated by APLTP. The results showed the sterilization efficiency against *E. coli* was about 10% after 5 min of exposure to UV generated by APLTP. UV germicidal action was optimal between 240 and 280 nm, few emission lines were visible in Figure 5. This is why the sterilization rate is low. The result is consistent with low disinfection efficiency of UV.

### 2.2. The Application of APLTP to Sterilize Fresh-Cut Cucumbers

The result of fresh-cut cucumber sterilization showed that the distance between the samples and electrode was 2.2 cm, the treatment voltage was 2.5 kV, the processing time was 5 min, and the sterilization efficiency against *E. coli* could reach 99.99%.

#### 2.2.1. Effect of APLTP Treatment on the Physicochemical Properties of Fresh-Cut Cucumber

Fresh-cut cucumber quality indicators include moisture content, soluble solids, pH, color, taste, shape and so on. The changes of the physicochemical properties of fresh-cut cucumber after APLTP treatment are shown in Table 1. The moisture content, soluble solids and pH value of fresh-cucumber after APLTP treatment did not change significantly (*p* > 0.05). These findings show that the water, sugar content and the acidity of fresh cucumber have been well maintained after APLTP treatment. Similar findings were reported by Won, Lee and Min [28], who found no significant differences for titratable acidity in control and cold-plasma-treated mandarins.

Relative conductivity is one of the important indicators of membrane permeability of fruits and vegetables. In the present study, we found that the relative conductivity was higher in treated cucumbers, indicating that the greater the permeability of the cell membrane, the more serious the cell damage [29]. Malondialdehyde (MDA) is the product of membrane lipid peroxidation. Accordingly, MDA is usually used as an important indicator of membrane lipid peroxidation to indicate the degree of lipid peroxidation and the strength of the damage due to adversity [30]. As shown in Table 1, the changes of relative conductivity and MDA content were not significant (*p* > 0.05). This finding shows that the effect of APLTP on the cell membrane of fresh-cut cucumbers can be neglected. Vc is an important indicator of the quality of the cucumber. The content of Vc in cucumber after APLTP treatment did not change significantly (*p* > 0.05), which indicated that APLTP can maintain the Vc content of cucumber well. Thus, plasma is a potent sterilizing agent and its treated process can preserve fresh-cut cucumber properties.

#### 2.2.2. Effect of APLTP Treatment on Sensory Indicators of Fresh-Cut Cucumber

Color plays a key role in food choice and is among the most important parameters for consumer acceptability. From the data presented in Table 2, the color parameters (brightness L *, red-green a *, blue-yellow b *) did not change significantly (*p* > 0.05) in treated fresh-cut cucumbers. These results are in agreement with those reported for cold-plasma-treated strawberries and cherry tomatoes, where changes in color among the control and treated fruits were found to be insignificant [31,32].

After plasma treatment, the content of (*E*,*Z*)-2,6-nonadienal decreased slightly with cucumber taste but not significantly. The decrease of the (*E*)-2-nonenal content reduced the unpleasant odor in cucumber. Also, the increase of hexanal and (*E*)-2-hexenal gave the cucumber a better fragrance. The increase in *cis* structure makes the refreshing aroma more concentrated. Accordingly, significant increase of (*Z*,*Z*)-3,6-nonadienal gave cucumber a more refreshing aroma. It is thus clear that the APLTP treatment caused a series of complex chemical changes in the flavoring components listed in Table 2, which led to the increase or decrease of the main aroma components and made the cucumber aroma better.

**Figure 6:**
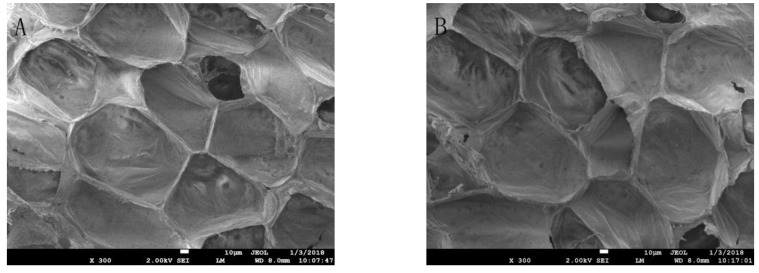
Scanning electron microscopy (SEM) images of cucumber pulp. (**A**) Control cucumber pulp cells; (**B**) Treated cucumber pulp cells.

The images shown in Figure 6A,B reveal that the control and treated cucumber pulp cells are intact and neatly arranged. In addition, the cell gap is tight and there is no wrinkle crimp. Also, there is no obvious perforation in the cell wall. These findings show that APLTP treatment does not damage the morphology of the cucumber pulp.

## 3. Discussion

In this study, we investigated the effectiveness and mechanism of action of the APLTP method as a sterilization tool against *E. coli* using a variety of techniques, which included SEM and FM analysis, protein leakage assay, and evaluation of cell membrane permeability. The results revealed that plasma treatment of the bacteria cells caused rupture of the cell wall and cell membrane, leakage of the cell content, and loss of the ability to reproduce and self-replicate. All these effects directly affected the metabolic function of the cell to achieve the bactericidal activity. Some previously reported research results regarding the bactericidal mechanism showed that temperature, UV light, high-voltage electric fields, charged particles and reactive oxygen species (ROS), as well as other external conditions, all play a role in the low-temperature plasma sterilization process [33,34,35]. Some of these agents act on the cell wall or penetrate the cell wall and cause irreversible damage to the interior of the bacteria. The reasons for such an effect include a series of physical and chemical reaction processes that eventually lead to cell death [36].

The microbial inactivation effect of the plasma treatment can be attributed to several synergistic mechanisms, including the generation of UV radiation, ozone, charged particles and ROS, in addition to other reactive species [34], which can damage microbial membranes, DNA and proteins [37]. The temperature during the APLTP treatment process is below 35 ± 1 °C, which is close to the optimal growth temperature of *E. coli*. This indicates that thermally-induced damage cannot be the sterilization mechanism. Another possible sterilization mechanism is UV radiation-induced damage, especially in the 200–280 nm wavelength range where DNA destruction is most effective, and which plays an important role in low-pressure plasma sterilization [38,39,40]. According to the data of optical emission spectroscopy, UV is only a minor contributor to *E. coli* inactivation, while excited argon atoms and OH are main responsible for the APLTP sterilization.

The effects of APLTP on the physicochemical properties and sensory quality indicators of fresh-cut cucumber were studied by analyzing the changes of moisture content, soluble solids, pH value, color, relative conductivity, MDA level, Vc content, aroma composition and microstructure. The results showed that there was no significant change in any of these indices (*p* > 0.05). APLTP can maintain water, sugar, acidity, Vc and color, and improve the aroma of the cucumber. Additionally, APLTP maintains cell membrane permeability and does not cause lipid oxidation. Furthermore, it was also found by SEM analysis that APLTP did not destroy the cell structure of fresh-cut cucumber pulp. Thus, APLTP may be used as an alternative to thermal pasteurization and may result in higher-quality fresh-cut fruits and vegetables.

## 4. Experimental Section

### 4.1. Sterilization Mechanism

#### 4.1.1. Experimental Apparatus

**Figure 7:**
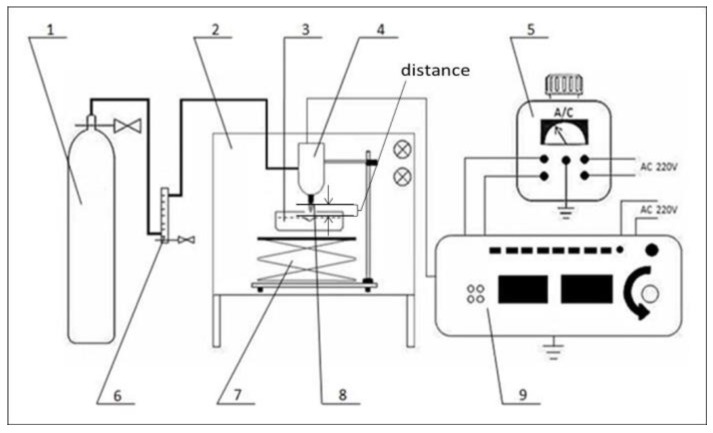
Schematic diagram of the experimental setup of the APLTP system. 1, argon cylinder; 2, super-clean worktable; 3, sample; 4, plasma electrode; 5, TDGC2-2 type contact voltage regulator; 6, LZB-10 float flow meter; 7, altitude controller; 8, plasma flame; 9, high frequency voltage AC power supply.

The schematic diagram of the experimental setup of the APLTP system is shown in Figure 7. The sample was placed in a super-clean worktable. Argon gas (99.99% purity) was used as the source of plasma and its flow rate was controlled by a float flow meter (LZB-10, Yuyao Yinhuan Flowmeter Co. Ltd., Yuyao, China). The distance between the samples and electrode can be controlled by adjusting the altitude controller. The discharge was driven by a high-frequency AC power supply, which can provide an output of maximal peak voltage of 2.5 kV. Based on the results from previous experiments on sterilization of fresh-cut cucumber, the distance between the samples and electrode was 2.2 cm, the treatment voltage was 2.5 kV, the processing time was 5 min, gas flow was 0.75 dm^3^∙min^−1^ and the sterilization efficiency against *E. coli* could reach 99.99%.

#### 4.1.2. Preparation of Inoculum

*E. coli* (No. E0005B) was obtained from Guangdong Huankai Microbial Sci. & Tech. Co., Ltd. (Guangzhou, China). *E. coli* was grown overnight in a sterilized Luria-Bertani (LB) broth in a shaker at 37 °C to prepare a bacterial suspension at a concentration of about 8 log CFU/mL. One mL of bacterial suspension was added into a 30-mm-diameter quartz petri dish and the dish was placed in a stage under the plasma jet.

#### 4.1.3. SEM Observation

The treated *E. coli* was washed three times with 0.1 M phosphate-buffered saline (PBS) and fixed twice with 2.5% glutaraldehyde and 1% osmium tetroxide for 12 h at 4 °C. Then, the fixed *E. coli* cells were dehydrated by an ethanol gradient (50%, 70%, 80%, 90% and 100%), washed with PBS and ultimately resuspended in PBS. In order to prevent surface charging by the electron beam, the treated *E. coli* cells were coated on the conductive adhesive, dried in a vacuum and sprayed with gold. Cell morphology was observed and photographed using a SEM (JEOL 7500F SEM, JEOL, Tokyo, Japan). Besides observing plasma-treated cells, untreated cells were used as control.

#### 4.1.4. FM Observation

The plasma-treated bacterial suspension was double stained with AO/PI for observation. First, 100 μL of reagent C was mixed with 900 μL of sterile deionized water to prepare the staining buffer solution. Second, the treated bacterial suspension was washed twice with 0.1 M PBS. Third, 500 μL of staining buffer solution was added to resuspend the cells. Fourth, 5 μL of AO and 10 μL of PI dyes were added sequentially. Subsequently, the mixture was washed with 0.1 M PBS after incubating in the dark at 4 °C for 10–20 min. Eventually, the cells were observed on a fluorescent microscope (LEICA TCS SP5 II FM, Leica Microsystems, Wetzlar, Germany). Besides observing plasma-treated cells, untreated cells were used as a control.

#### 4.1.5. Determination of Conductivity

A volume of 2 mL of treated bacterial suspension was centrifuged at 5000 rpm for 10 min. Supernatants were diluted 20-fold and tested for conductivity with a conductivity meter (FE38 conductivity meter, Mettler-Toledo Instrument Co. Ltd., Shanghai, China). Besides testing plasma-treated cells, untreated cells were tested as a control. At the same time, the change of conductivity of the cell-free LB nutrient broth before and after treatment was measured. The experiment was repeated 3 times and the data were averaged.

#### 4.1.6. Determination of Protein Concentration

Protein content was determined using the Coomassie Brilliant Blue method. *E. coli* supernatant (0.1 mL) was accurately pipetted into a 10 mL stoppered test tube, then Coomassie Brilliant Blue G250 (5 mL) stain was added into test tube, and stood for 2 min after mixing. The absorbance was measured at a wavelength of 595 nm using a 10-mm-thick cuvette, and the protein content of the corresponding sample was determined from the standard curve.

#### 4.1.7. Optical Emission Spectroscopy

The optical emission spectra were recorded on the AvaSpec-2048-8-RM spectrometer (Avantes, Eerbeek, The Netherlands) equipped with gratings of 2400 grooves∙mm^−1^, and the distance from an optical fiber to the nozzle of plasma jet is the same as the distance from sample to the nozzle of plasma jet.

### 4.2. The Application of APLTP for Sterilization of Fresh-Cut Cucumber

#### 4.2.1. APLTP Treatment

Fresh cucumbers were purchased from the local supermarket, sterilized with 70% ethanol in order to reduce the background microbial load, and then washed with sterile deionized water to remove any remaining ethanol residue. The sterilized cucumber was sliced with a sterile knife in a sterile operating room. *E. coli* (1 mL) was inoculated onto 25 g of fresh-cut cucumbers and then dried for 30 min in a sterile operating room. Eventually, the fresh cucumber was treated under a plasma jet. The plasma discharge was produced at a voltage of 2.5 kV and a frequency of 33 kHz. In the experiments, the flow rate of the argon was fixed at 0.75 dm^3^∙min^−1^. Slices of fresh-cut cucumbers were put in a petri dish. The distance between the cucumber sample and the plasma outlet was 2.2 cm. To ensure that the entire surface was treated by the plasma, the petri dish was moved regularly. After 5 min APLTP treatment, fresh-cut cucumber slices were transferred into a sterilized bag with 10 mL PBS, and treated for 10 min at the highest speed in a homogenizer. 100 μL of cell suspension of the PBS were spread uniformly over LB nutrient agar plates, and then incubated at 37 °C for 24 h for CFU counting. The inactivation rate is defined as (1 − CFU_treated_/CFU_control_) × 100%.

#### 4.2.2. Influences of the Plasma Treatment on the Physicochemical Properties of the Cut Cucumber

The moisture content was measured according to GB 5009.3-2016 National Food Safety Standard. Fresh-cut cucumber were squeezed and homogenized. Aliquots of the homogenized material were analysed for SSC and pH. SSC (PAL-Fu refractometer, Atago Scientific Instrument Co. Ltd., Guangzhou, China) was analysed by measuring the refractive index with a digital refractometer; pH was measured with a pH meter (pHS-3C pH meter, Qi Wei Co. Ltd., Hangzhou, China); MDA content was measured by the thiobarbituric acid colorimetry method; Vc was determined by the molybdenum blue colorimetry method. Triplicate measurements were taken for each sample and the results were averaged.

To determine the relative conductivity, cucumbers were cut into slices with the same size and uniform thickness. Two grams of cucumber slices were accurately weighed and immersed in 50 mL distilled water for 2 h. Conductivity was measured with a conductivity meter (FE38 conductivity meter, Mettler-Toledo Instrument Co. Ltd., Shanghai, China). Then, the cucumber slices were heated and boiled for 20 min, and the conductivity was measured after cooling. The measurement was performed in triplicate. The relative conductivity was calculated according to Equation (1).

(1) $$R\left(\%\right)=\frac{R_{1}}{R_{2}}\times 100$$

Determination of the aroma components—extraction of volatile components: 6 mL of minced cucumber sample was filtrated into a 20 mL headspace bottle with 1.5 g NaCl. The headspace bottle was tightened and placed in a water bath at 50 °C for 5 min with shaking. The aged ZZ-SPME-06 extraction head was allowed to adsorb for 40 min, and then the extraction head was inserted into the gas chromatography system (5975B/6890N GC-MS, Agilent Technologies, Santa Clara, CA, USA) inlet for desorption for 20 min. Gas chromatography conditions: HP-5MS column (30 m × 320 μm, 0.25 μm); inlet temperature was 260 °C; carrier gas was 99.999% He; splitless injection. The temperature program was started at 40 °C for 2 min, and then ramped to 60 °C for 1 min at 4 °C/min, and then ramped to 150 °C for 0 min at 2 °C/min, and finally raised to 210 °C for 5 min at 10 °C/min. The column was baked for 5 min at 260 °C. Mass spectrometry conditions: The scope of the scan was 50–550 amu, the threshold was 150 and the sampling frequency was 5 scans/s. Qualitative analysis of the chromatographic peaks: The mass spectral data of each component was automatically searched in the NIST2002 standard library. The results of both the positive and the negative matching degrees higher than 800 only are reported. The quantitative analysis used the peak area normalization method.

#### 4.2.3. The Influences of the Plasma Treatment on the Sensory Indicators of the Cut Cucumber

Determination of the flesh color: The color difference was measured using a colorimeter (CR-400 colorimeter, Konica Minolta Company, Tokyo, Japan), and the measured parameters were L * value, a * value and b * value. Each sample was analyzed in triplicate.

The determination of the flesh morphology: The treated cucumber slices were fixed with 4% glutaraldehyde at 4 °C for 6 h, rinsed three times with PBS and then fixed with 1% osmium tetroxide at 4 °C for 2 h followed by three rinses with PBS. The samples were dehydrated with a 10%, 30%, 50%, 70%, 80%, 90% and 95% alcohol gradient and then alcohol was removed stepwise with 25%, 50% and 75% *t*-butyl alcohol. Eventually, the cucumber slices were immersed in pure *t*-butyl alcohol and dried for about 3 h. Subsequently, the slices were subjected to the conductive plastic spray treatment, and the samples were examined by SEM.

### 4.3. Statistical Analysis

Statistical analysis was performed using the SPSS 19.0 software (SPSS Inc., Chicago, IL, USA). The data collected following APLTP treatment were subjected to analysis of variance (ANOVA). Means were compared according to Fisher’s least-significant difference method at the 0.05 level. All the results represent the average of three separate experiments.

## Figures and Tables

**Table 1:** Effect of APLTP treatment on the physicochemical properties of fresh-cut cucumber.

Indicators	Control	Treated
Moisture content (%)	95.135 ± 0.05 ^a^	95.153 ± 0.05 ^a^
Vc (mg/100 g)	20.25 ± 0.10 ^a^	20.27 ± 0.10 ^a^
Relative conductivity (%)	9.32 ± 0.10 ^a^	9.35 ± 0.10 ^a^
MDA (μmol/L)	0.00121 ± 0.00011 ^a^	0.00125 ± 0.00012 ^a^
Soluble solids (Brix)	3.6 ± 0.10 ^a^	3.7 ± 0.12 ^a^
pH	6.02 ± 0.10 ^a^	6.05 ± 0.10 ^a^

^a^ No significant difference (*p* > 0.05).

**Table 2:** Effect of APLTP treatment on sensory indicators of fresh-cut cucumber.

	Indicators	Control	Treated
Aroma components	(*E*,*Z*)-2,6-Nonadienal	42.53 ± 2.54 ^a^	40.61 ± 1.11 ^a^
	(*E*)-2-Nonenal (%)	12.54 ± 0.57 ^a^	11.54 ± 0.58 ^a^
	Hexanal (%)	9.71 ± 0.54 ^a^	10.64 ± 1.33 ^a^
	(*E*)-2-Hexenal (%)	4.42 ± 0.28 ^a^	4.72 ± 0.39 ^a^
	(*Z*,*Z*)-3,6-Nonadienal (%)	14.09 ± 0.17 ^b^	14.59 ± 0.32 ^a^
Color parameters	L *	63.78 ± 0.25 ^a^	63.89 ± 0.23 ^a^
	a *	−6.07 ± 0.10 ^a^	−5.98 ± 0.15 ^a^
	b *	16.61 ± 0.10 ^a^	16.63 ± 0.13 ^a^
	△E	0	0.73 ± 0.25

^a^ No significant difference (*p* > 0.05). ^b^ Significant difference (0.01 < *p* < 0.05).
